# Speciation Characteristics and Ecological Risk Assessment of Heavy Metals in Municipal Sludge of Huainan, China

**DOI:** 10.3390/molecules26216711

**Published:** 2021-11-05

**Authors:** Mu You, Yunhu Hu, Yule Yan, Jie Yao

**Affiliations:** 1 National Center of Coal Chemical Products Quality Supervision & Inspection (Anhui), Huainan 232001, China; youmu@ustc.edu.cn (M.Y.); yaojie@aust.edu.cn (J.Y.); 2 Key Laboratory of Bioresource and Environmental Biotechnology of Anhui Higher Education Institutes, Huainan Normal University, Huainan 232001, China; huyunhu@ustc.edu.cn

**Keywords:** heavy metals, sludge, speciation characteristics, ecological risk assessment

## Abstract

In order to fully understand the morphological characteristics and pollution status of heavy metals in the dewatered sludge of Huainan Municipal sewage treatment plant, the physical and chemical properties were analyzed, and the content and occurrence forms of heavy metals (As, Cu, Zn, Pb, Cd, Cr, and Ni) in the sludge were studied using the geological accumulation method (Igeo), risk assessment coding method (RAC), and potential ecological risk index method to evaluate the ecological risk. The results showed that the municipal sludge in Huainan was rich in nutrients, with good prospects for agricultural utilization. There were differences in the morphological distributions of different heavy metals. The *Igeo* values for Ni, As, Cr, and Pb were below 0. The results of RAC indicated that the risk level of Cr in sludge was a low risk, and those of other heavy metals were moderate risks. The potential ecological risk of Cd had the highest potential ecological risk, and the other six metals were of low ecological risk. This conclusion can provide basic data and a theoretical reference for the comprehensive utilization of sludge in sewage treatment plants.

## 1. Introduction

With the rapid development of economy and the advancement of urbanization, the discharge of urban domestic sewage is increasing, the sewage treatment capacity also increases [1]. Municipal sludge is a solid, semi-solid, or liquid waste produced in the process of urban sewage treatment, which is composed of a variety of bacterial micelles and various organic and inorganic substances adsorbed by them [2,3]. The sludge production in the process of sewage treatment continues to increase significantly, and the safe disposal and reuse of sludge has attracted extensive attention [4,5]. 

There is a lot of organic matter and rich plant nutrients such as nitrogen, phosphorus, and potassium in sludge, which is a good fertilizer and soil conditioner [6]. Land use of sludge is not only an important method of urban sludge disposal but also the fundamental way out for sludge disposal in the future [7,8]. It is of great significance for the sustainable development of cities and agriculture [1]. Sludge also contains a certain amount of heavy metals, which are easy to accumulate, difficult to degrade, and harmful [9]. 

During the agricultural process, the heavy metals in sludge will exist in the environment for a long time and accumulate continuously after entering the soil, resulting in certain potential ecological and health risks [10,11,12,13]. Heavy metals have always been the key factor limiting the effective agricultural use of sludge, and sludge is the main source of heavy metal pollution in soil [14]. Therefore, it is of great significance to obtain the environmental effects of heavy metals in sludge and to evaluate its potential ecological risks before sludge can be effectively utilized [15].

The ecological risk of heavy metals in municipal sludge is related to the types, concentrations, and chemical fractionation of heavy metals [16]. For environmental effects, the content of heavy metal can indicate the overall level and mobility of metals in sludge, but the bioavailability and ecotoxicity of heavy metals largely depend on their specific chemical form or chemical combination mode [17]. The behavior of heavy metals in sludge is not only related to its total amount but also depends on its chemical form [14]. Therefore, the speciation analysis of heavy metals in sludge is particularly important. Continuous extraction is an important method to study the occurrence state of heavy metals, which is of great significance to the assessment of soil environmental risk.

Different chemical speciation of heavy metals can be determined with selective sequential extraction analysis, and different extractants can be used to separate and extract different components of the same heavy metal with different chemical reagents and conditions [18]. The most widely used sequential extraction method was proposed by Tessier et al. [19]: a five-stage continuous extraction method for the analysis of bound state of soil heavy metals, which divided heavy metals into exchangeable state, carbonate-bound state (acid soluble state), iron-manganese-oxide-bound state (reducible state), organic-matter-bound state (oxidizable state), and residue state. The organic-bound state and the residual state are not easily absorbed by organisms in the environment and belong to the stable state. The exchangeable state, carbonate-bound state, and Fe-Mn oxide binding state are easily absorbed directly or indirectly and belong to the unstable state [20]. Many scholars have conducted research on heavy metal pollution using the Tessier sequential extraction method in samples of soils [21], sediments [22], and sewage sludge [15,23].

The evaluation of heavy metal pollution in sludge can provide a basis for pollution prevention and control and the resource utilization of sludge. Many studies have proposed a variety of heavy metal pollution assessment methods, such as the geological accumulation index (index of geo accumulation, Igeo) [24], Nemerow aggregation index (NPI) [25], risk assessment code (RAC) [26,27], potential ecological risk factor (ER), and risk index (RI) [28]. Different evaluation methods are widely used to evaluate various environmental samples. As each evaluation method is different, the results are also different. Therefore, multiple methods should be selected for a comprehensive evaluation during ecological evaluation.

Huainan is an important energy city. With the acceleration of urbanization, the amount of sludge, as a solid waste produced in the process of sewage treatment, has increased sharply; however, there have been limited reports on the content of nutrients and heavy metals in urban sludge. In this study, four representative and well operated urban sewage treatment plants in Huainan were selected for investigation and sampling, the nutrient characteristics and heavy metal pollution status of municipal dewatered sludge in Huainan were analyzed, and the occurrence characteristics of heavy metals in sludge were measured using the Tessier sequential extraction method. The contamination degree and risk of heavy metals were evaluated with the aid of the geo-accumulation index (*I*geo), and the risk assessment code (RAC) and potential ecological risk index (RI) were used to evaluate the potential harm of seven heavy metals in sludge to the ecological environment in Huainan City, which will provide a scientific basis for the resource disposal of sludge in Huainan City.

## 2. Materials and Methods

### 2.1. Sample Collection

**Figure 1:**
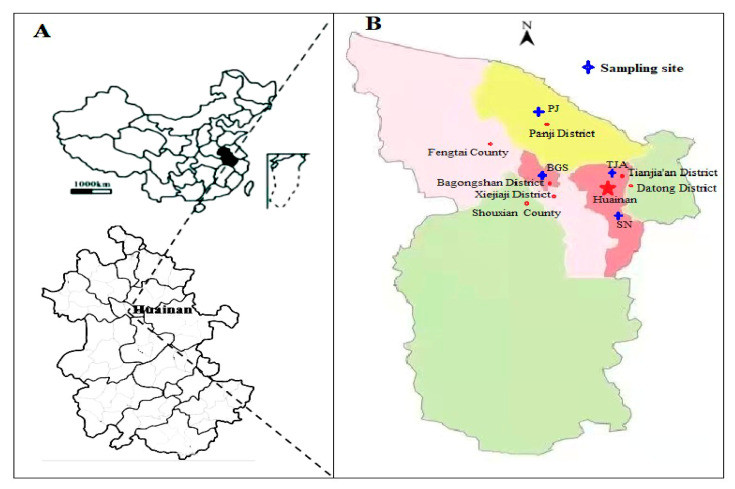
Sampling sites of municipal sewage treatment plants in Huainan.

Huainan is located in east of China and north central of Anhui, which consists of five districts and two counties with a population of 3.0 × 10^6^ and covers 5533 km^2^. Huainan is the energy capital of China, the industrial granary of East China and an important industrial city in Anhui Province. Wet sludge samples after dehydration were collected from municipal sewage treatment plants in different locations in the urban area of Huainan City (Figure 1): TJA (Tianjiaan), SN (Shannan), PJ (Panji), and BGS (Bagongshan). The details of these plants are indicated in Table 1. After mixing evenly, the samples were sealed in a polyethylene bag under freezing conditions and brought to the laboratory as soon as possible. The samples were air-dried at room temperature, the impurities were removed, the samples were sampled by the quartering method, grinded through a 100-mesh sieve, sealed, and stored in a drying oven for testing. 

### 2.2. Sample Analysis

For the determination of the physical and chemical properties, nutritional indexes, and heavy metal content of sludge samples, we referred to the inspection method for sludge from a municipal sewage treatment plant (CJ/T 221-2005) [29]. The pH of the sludge samples was determined by the glass electrode method; Organic matter was determined by potassium dichromate oxidation spectrophotometry, the total nitrogen was determined by alkaline potassium persulfate digestion ultraviolet spectrophotometry, the total phosphorus was determined by sodium hydroxide melting molybdenum antimony resistance spectrophotometry, and the total potassium was determined by atmospheric pressure digestion flame atomic absorption spectrophotometry.

The total contents of Cr, Cu, Pb, Mn, Ni, and Zn in the sewage sludge samples were analyzed by inductively coupled plasma mass spectrometry (ICP-MS, 820-MS, Varian, Palo Alto, CA, USA) after the pretreated samples were digested by a microwave digester (Multiwave PRO, Antonpa, Australia) with the tri-acid digestion (HNO_3_-HF-HClO_4_) method. In this study, reference materials for soil composition analysis (GBW 07449) and stream sediment composition analysis (GBW 07307a) were used. Three parallel samples were set for each batch of standard reference materials, and the measured recovery was 90–110%. Parallel samples, standard addition, and recovery were adopted for quality control. The fraction of heavy metals was determined using a five-step sequential extraction procedure proposed by Tessier et al. (1979) [19]. All samples were controlled by blank sample test, parallel sample test, and standard recovery test. During the extraction, metals were classified into analysis of the residual fractions.

Step one: Exchangeable (F1). 8 mL MgCl_2_ solution with a concentration of 1 mol·L^−1^ was added to 1.00 g dry sludge soil sample. The samples were oscillated at 250 r·min^−1^ for 1 h in a JDWZ-2012 constant temperature and humidity culture oscillator at 25 °C (pH = 7.0). Centrifugation was executed at 5000 r·min^−1^ for 10 min, and the supernatant was placed in a 50 mL constant volume colorimetric tube to be measured.

Step two: Bound to Carbonates (F2). 8 mL of 1 mol·L^−1^ NaAc solution was added to the sediment of the first step, and the solution was oscillated at 250 r·min^−1^ for 5 h at 25 °C (pH = 5.0). After centrifugation at 5000 r·min^−1^ for 10 min, the supernatant was placed in a constant volume colorimetric tube for testing.

Step three: Bound to Fe-Mn oxides (F3). 20 mL of 0.04 mol·L^−1^ NH_2_OH·HCl solution (pH = 2.0) was added to the residue of the second step. After a water bath at 96 °C for 6 h, the residue was oscillated at 250 r·min^−1^ for 1 h and centrifuged at 5000 r·min^−1^ for 10 min. We placed the supernatant in a colorimetric tube and waited for measurement.

Step four: Bound to Organic Matter (F4). The residue of the third step was added to 3 mL of 0.02 mol·L^−1^ HNO_3_ solution (pH = 2.0) and 5 mL of 30% (V·V^−1^) H_2_O_2_ water bath at 85 °C for 2 h, and was shaken for 1 h at 250 r·min^−1^. Then, we added another 3 mL of H_2_O_2_ to the solution, and the oscillation continued for 3 h. Subsequently, we added 5 mL NH_4_Ac solution and shook for 30 min. We centrifuged the supernatant to be measured.

Step five: Residual (F5). The remaining 0.50 g residue was added to 10 mL water, HNO_3_ and HCl mixed solution (volume ratio of water, HNO_3_ and HCl = 4:1:3). The residue was oscillated in a 100 °C water bath at 250 r·min^−1^ for 2 h and tested after demonstrating a constant volume of solution.

### 2.3. Contamination Degree and Risk Analysis

#### 2.3.1. Geo-Accumulation Index (Igeo) Method

The Geo-accumulation index (Igeo) was proposed by Muller [30] in 1969. It is a method to evaluate the pollution degree of heavy metals in sludge. Compared with other similar evaluation methods, the Geo-accumulation index method considers human pollution factors, environmental geochemical background values, and possible changes in background values caused by human activities. The formula for calculation is as follows:

(1) $$lgeo=log_{2}\left[C_{i}/kB_{i}\right]\;$$

where $C_{i}$ is the measured mass fraction of heavy metal *i* in sewage sludge;$\;B_{i}$ is the preindustrial geochemical background value of heavy metal *i*, and *k* is 1.5. The geometric mean values of heavy metals in the surface soil of Anhui Province, China were used as background reference values. The background contents of As, Cu, Zn, Pb, Cd, Cr, and Ni are 17.7, 63.7, 29.7, 0.0696, 39.4, and 15.8 mg/kg [31], respectively. The accumulation index and pollution degree classification of heavy metals are shown in Table 2.

#### 2.3.2. Mobility and Availability of Heavy Metals Assessment Method

RAC ecological risk assessment method [32] is a potential risk assessment method of heavy metals based on heavy metal speciation analysis. It is a useful risk characterization method of heavy metals in sediments. It is characterized by the mass percentage of carbonate-bound/ion exchangeable heavy metals in the total amount of heavy metals and is calculated using the following formula:

(2) $$RAC=\frac{F_{1}+F_{2}}{C_{t}}*100\%$$

where *F*_1_ is the concentration of metal in exchangeable fraction, *F*_2_ is the concentration of metal in the carbonate fraction, and *C_t_* is the total concentration of metal in five fractions. The RAC risk assessment scale is indicated in Table 2.

#### 2.3.3. Potential Ecological Risk Assessment of Heavy Metals

The potential ecological risk assessment method, proposed by Hakanson [33], quantitatively divides the potential ecological hazard degree by measuring and analyzing the pollutant content in the sample, which can reflect the comprehensive impact of various heavy metals on the ecological environment, and has been proven to be a relatively fast, simple, and standard method for dividing the pollution degree of heavy metals and potential ecological risks. It is widely used in the ecological risk assessment of heavy metals in sediment, soil, and sludge. It is determined using the following formulas:

(3) $$E_{r}^{i}=T_{r}^{i}C_{f}^{i}$$

(4) $$C_{f}^{i}=C_{0}^{i}/B_{n}^{i}$$

(5) $$RI=\sum E_{r}^{i}$$

where $E_{r}^{i}$ is the potential ecological risk coefficient of heavy metal *i*, $T_{r}^{i}$ is the toxicity coefficient of heavy metal *i* that reflects the toxicity, pollution levels, and sensitivity of the environment; the toxicity coefficients of As, Cd, Cr, Cu, Pb, Zn, and Ni are 10, 30, 2, 5, 5, 1, and 5, respectively. $B_{n}^{i}$ is the reference background value of the heavy metal [34]. $C_{0}^{i}$ is the concentration of heavy metal *i* (total content). RI is the sum of all heavy metal risk factors. The potential ecological risk degree classification of heavy metals is also shown in Table 2.

## 3. Results and Discussion

### 3.1. Physical and Chemical Characteristics of Sludge

The physicochemical characteristics parameters of sludge from Huainan Municipal sewage treatment plant are shown in Table 3. The pH of dewatered sludge is between 6.54 and 7.16, which presents weak alkalinity. The moisture content of sludge is 78.16%–82.341%. According to the disposal of sludge from municipal wastewater treatment plants: for the quality of sludge used in agriculture (CJ/T 309-2009), the physical indicators require that the water content is less than 60% and that the pH is in the range of 5.5–9.0. It can be seen that the pH and moisture content of sludge in each sewage treatment plant meet the standard requirements. The concentration of organic matters ranged from 287.69 to 352.46 g/kg. The concentrations of TN, TP, and TK in the sludge were distributed in the ranges of 22.54–32.16, 6.92–10.17, and 6.96–7.51 g/kg, separately. The results meet the requirements of organic matter content ≥ 200 g/kg, nitrogen + phosphorus + potassium content ≥ 30 g/kg [35]. The average content of organic matter in plant sludge is 320.21 g/kg, and the average content of nitrogen + phosphorus + potassium is 42.30 g/kg, which meets the specified indexes of nutrition. The Municipal Sludge in Huainan city is an organic fertilizer with high organic matter, high nitrogen, and high phosphorus content, which has a good prospect of agricultural utilization.

### 3.2. Content Characteristics of Heavy Metals

The total contents of seven heavy metals in Huainan Municipal Sludge ranged from 1011.38 mg/kg to 1112.05 mg/kg (Table 4). According to the average value, the total heavy metal content of sludge from different sewage plants is BGS > TJA > SN > PJ. It was found the average concentrations of heavy metals ranked in the following order: Zn (638.99 mg/kg) > Cu (240.33 mg/kg) > Cr (65.64 mg/kg) > Ni (52.42 mg/kg) > Pb (43.71 mg/kg) > As (10.31 mg/kg) > Cd (2.57 mg/kg). Comparing the pollutant discharge standard for urban sewage treatment plants (GB18918-2002) and the heavy metal content of sludge in different regions reported in the literature (Table 4), the heavy metal content of municipal sludge in Huainan is at a low level, and the compliance rate reaches 100%.

Literature studies [8,14,36,37] showed that Zn, Cu, Cr, and Pb are the main heavy metal pollution components in municipal sludge in major cities in China, which is similar to in this study. The total amount of heavy metals is lower than the national average level [36] and is significantly lower than that of economically developed cities, such as Guangzhou [14] and Beijing [37]. This may be due to the diversion of domestic sewage and industrial wastewater in Huainan City, which reduces the heavy metal pollution in municipal sewage. According to the requirements of sludge disposal standard for the heavy metal content of class A agricultural sludge and landscaping sludge in acidic soil, we found that the heavy metal content of Huainan municipal sludge was lower than the standard limit, thus, showing a great potential for resource utilization.

### 3.3. Speciation Patterns of Heavy Metals

**Figure 2:**
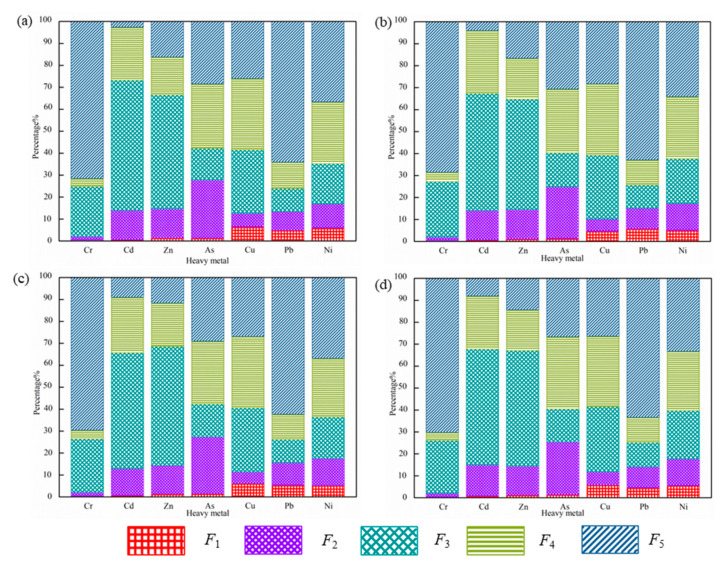
Chemical speciation of heavy metals in (**a**) TJA (Tianjiaan) (**b**) SN (Shannan) (**c**) PJ (Panji) and (**d**) BGS (Bagongshan).

Among the five fractionations of heavy metals, the residual fraction is a relatively stable phase that is difficult to migrate and transform under general conditions. It is considered that there is no risk of pollution to the environment under natural conditions, and the toxicity is the smallest. The acid exchangeable phase (F1), oxidizable phase (F2), and reducible phase (F3) are extractable phases and can be released under certain environmental conditions, absorbed by organisms, indicating the high mobility of heavy metals and have potential harm [14,17]. Various geochemical fractions of different heavy metals in the sludge are depicted in Figure 2. The morphological distribution of heavy metals in the sludge of the Huainan municipal sewage treatment plant is clearly different due to the different types of heavy metals. The mean contents of heavy metals in different fractions followed the orders:

Cr: Residual > Reducible > Organic > Carbonate > Exchangeable
Ni: Residual > Organic > Reducible > Carbonate > Exchangeable
Pb: Residual > Organic > Reducible > Carbonate > Exchangeable
Cd: Reducible > Organic > Carbonate >Residual > Exchangeable
Cu: Organic > Reducible > Residual > Exchangeable > Carbonate
Zn: Reducible > Organic > Carbonate > Residual > Exchangeable
As: Organic > Residual > Carbonate > Reducible > Exchangeable

The occurrence form of Cr in sludge was the residual phase (F5), accounting for 69.91%, which mainly existed in the residual state, followed by the oxidizable state (F3, Fe-Mn oxides), and reducible state (F2, Carbonate). It is difficult to release under natural conditions and is not easily biologically utilized due to its limited mobility. It is roughly the same as different occurrence forms of contents of Cr by comparison with the sludge of Bengbu City along the Huaihe River [34]. Cd in sludge mainly existed in the reducible phase accounting for 54.47% and has strong potential mobility. Studies have shown that the reducible state mainly reflects the environmental pollution caused by human activities, indicating that the main source of Cd in urban sludge is human activities [40].

The proximity of different forms of As indicates that As was evenly distributed in each phase of sludge. The mass fractions of Pb and Ni in sludge from large to small were the residual phase, organic-bound phase, iron-manganese-oxide-bound phase, carbonate-bound phase, and exchangeable phase, which are basically the same as the high stable state contents of Pb and Ni in sludge of many municipal sewage treatment plants in China.

Cu in municipal sludge mainly existed in the form of the oxidation state accounting for 31.53% with relatively strong stability and weak bioavailability and mobility. Zn mainly existed in the iron-manganese-oxide-bound state, which accounts for a large proportion (accounting for 52.22%). The unstable states of Zn and Cu in the sample sludge were the highest among the seven heavy metals, and the potential environmental risk is large. However, referring to the sludge agricultural standard, they were all within the standard value range, and thus they can be used safely.

### 3.4. Ecological Risk Assessment of Heavy Metal Pollution in Sludge

#### 3.4.1. Risk Assessment by the Geo-Accumulation Index

**Figure 3:**
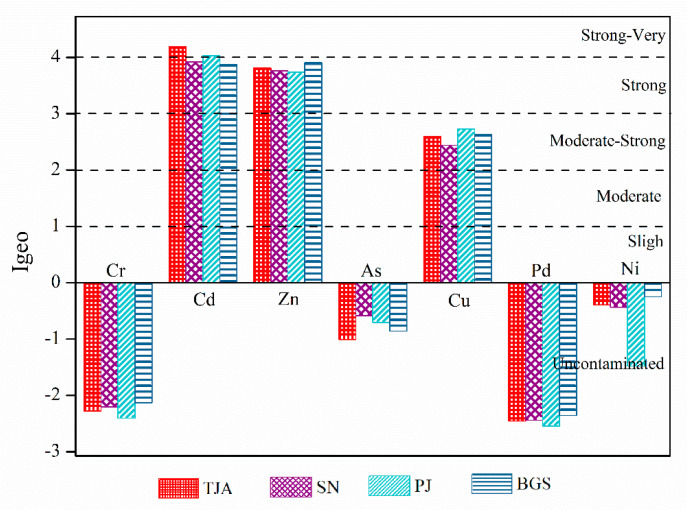
The *Igeo* results for the heavy metals in the sludge. TJA (Tianjiaan), SN (Shannan), PJ (Panji), and BGS (Bagongshan).

The contamination degree of heavy metals was assessed by the Geo-accumulation method (*Igeo*). The results are listed in Figure 3. The order of pollution degree was Cd > Zn > Cu > Ni >As > Cr > Pb. The pollution degree of Cd was the highest in the seven heavy metals, and the pollution level was at the strong contaminated grade. Zn was the second-highest pollution heavy metal, and the pollution level was moderate-strong. The pollution level of Cu was at the medium contaminated grade. The *Igeo* values for Ni, As, Cr, and Pb in the four sewage sludge samples were below 0. This indicates that the pollution levels of these four heavy metals in sewage sludge were not found.

#### 3.4.2. Mobility and Availability of Heavy Metals Assessment

**Figure 4:**
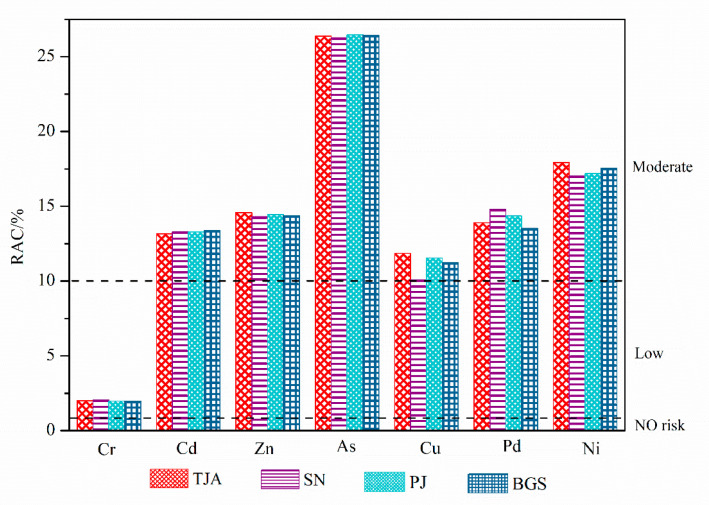
The RAC results for the heavy metals in the sludge. TJA (Tianjiaan), SN (Shannan), PJ (Panji), and BGS (Bagongshan).

Heavy metals in sludge are bound to different fractions with different strengths in changes of mobility and availability. The RAC is mainly used to evaluate the mobility and availability of the exchangeable/acid-soluble fraction-bound metals. The results of the RAC index are reported in Figure 4. Among the sludge samples from four municipal sewage treatment plants, the average values of RAC of seven heavy metals are As (26.38%) > Ni (17.42%) > Zn (14.42%) > Pb (14.14%) > Cd (13.28%) > Cu (11.18%) > Cr (1.99%), which also represents the order of effectiveness and environmental risk of seven heavy metals to a certain extent. The risk level of Cr in sludge is low risk, and that of other heavy metals is moderate risk.

#### 3.4.3. Potential Ecological Risk Assessment of Heavy Metals

**Figure 5:**
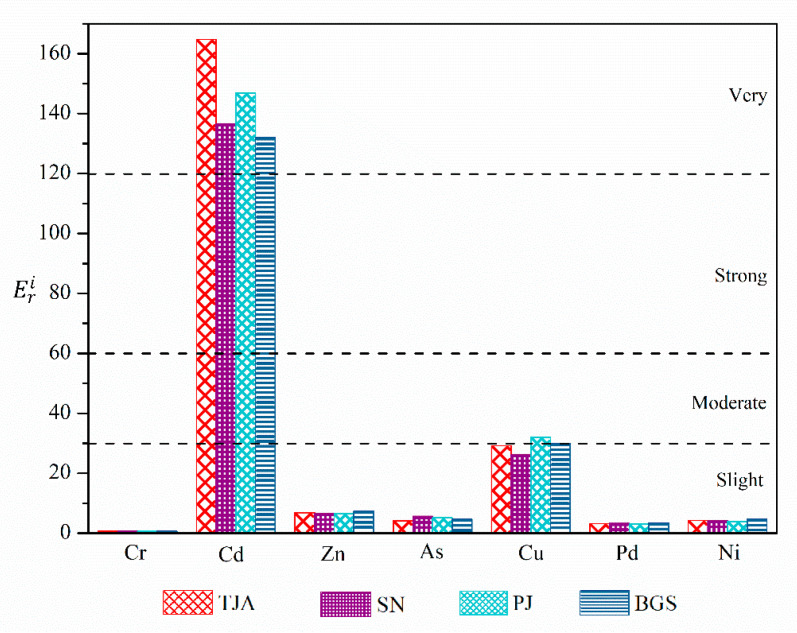
The potential ecological risk for the heavy metals in the sludge. TJA (Tianjiaan), SN (Shannan), PJ (Panji), and BGS (Bagongshan).

The Hakanson coefficient method was used to evaluate the potential ecological risk of heavy metals in sewage sludge. We calculated the $E_{r}^{i}$ value of a single heavy metal in each sewage plant and then calculated the average value of the corresponding heavy metals. The assessment results of heavy metals in the four municipal sewage treatment plants are presented in Figure 5. The order of the average value $E_{r}^{i}$ of heavy metals was Cd(145.04) > Cu(29.32) > Zn(6.81) > As(4.89) > Ni(4.25) > Pb(3.22) > Cr(0.66). Cd had the highest potential ecological risk, which belongs to high ecological risk, and the other six metals were low ecological risk. The comprehensive ecological risk RI value of municipal sewage treatment plant ranged from 182.62 to 213.06 with an average value of 194.18, belonging to the moderate risk level. In other words, if the sludge is directly discharged into the environment without any pretreatment, the safety of the ecological environment will be threatened. Furthermore, the high ecological risk of sludge is mainly caused by cadmium [17].

## 4. Conclusions

In this study, the contents of Cu, Pb, CD, Cr, Ni, Zn, As, and Hg in the dewatered municipal sludge of four sewage treatment plants in Huainan were measured, and the ecological risk was evaluated. We found that the sludge from the Huainan Municipal sewage treatment plant had good prospects for agricultural utilization. The distribution of different heavy metal fractions was essentially uniform. The pollution level of Cd was higher than other heavy metals and had the highest potential ecological risk. From the speciation analysis of heavy metals, the proportion of bioavailable states of As and Cd was high. Therefore, when considering the agricultural use of the sludge, special attention should be paid to the stabilization treatment of heavy metals.

## Figures and Tables

**Table 1:** Detailed information of the four wastewater treatment plants.

Sample	Capacity/ (m^3^·day^−1^)	Wastewater Treatment Technology	Sewage Type	Sludge Dewatering Technique
TJA (Tianjiaan)	1.00 × 10^5^	Oxidation ditch, SBR	Domestic sewage	Belt filter
SN (Shannan)	5.00 × 10^4^	Oxidation ditch, SBR	Domestic sewage	Rotary press filter
PJ (Panji)	4.00 × 10^4^	Oxidation ditch, SBR	Domestic sewage	Belt filter
BGS (Bagongshan)	1.00 × 10^5^	A^2^/O	Domestic sewage	Belt filter

**Table 2:** Grading standards of potential ecological risk.

Geo-Accumulation Index (Igeo)			Classification of RAC Risk Assessment		Grading Standards of Potential Ecological Risk		
Geo-Accumulation index (Igeo)	Degree	Risk Level	Evaluation Index Range	Risk Level	$E_{r}^{i}$	*RI*	Risk Level
Igeo < 0	*0*	Uncontaminated	RAC < 1%	No risk	$E_{r}^{i}$ < 30	*RI* < 100	Slight
0 ≤ Igeo < 1	1	Slight	1% < RAC ≤ 10%	Low	30 ≤ $E_{r}^{i}$ < 60	100 ≤ *RI*< 200	Moderate
1 ≤ Igeo < 2	2	Moderate	10% < RAC ≤ 30%	Moderate	60 ≤ $E_{r}^{i}$ < 120	200 ≤ *RI*< 400	Strong
2 ≤ Igeo < 3	3	Moderate–Strong	30% < RAC ≤ 50%	High	120 ≤ $E_{r}^{i}$ < 240	*RI* ≥ 400	Very strong
3 ≤ Igeo < 4	4	Strong	RAC > 50%	Extremely high	$E_{r}^{i}$ ≥ 240		Extremely strong
4 ≤ Igeo < 5	5	Strong–Very strong					
Igeo ≥ 5	6	Extremely Strong					

**Table 3:** Physicochemical characteristics of the sewage sludges.

Sample	PH	Moisture Content/ (%)	EC/ (μs·cm^−1^)	Organic Matter/ (g·kg^−1^)	TN/ (g·kg^−1^)	TP/ (g·kg^−1^)	TK/ (g·kg^−1^)	TN+TP+TK/ (g·kg^−1^)
TJA	6.83	78.16	980	324.15	24.13	6.92	7.23	38.28
SN	6.92	82.12	1200	287.69	22.54	8.14	7.51	38.19
PJ	7.16	79.32	1300	316.53	27.18	10.17	6.96	44.31
BGS	6.54	82.34	1060	352.46	32.16	9.18	7.08	48.42
Mean value	6.86	80.49	1135	320.21	26.50	8.60	7.20	42.30
CJ/T 309-2009	5.5–9.0	≤60	-	≥200	-	-	-	≥3

**Table 4:** Comparison of the heavy metal contents in municipal sludge from the literature (mg/kg).

	As	Cd	Cr	Cu	Ni	Pb	Zn	Literatures
TJA	8.76	2.82	67.92	236.52	53.68	44.53	652.36	
SN	12.13	2.37	64.53	218.79	50.16	43.62	634.18	
PJ	10.87	2.69	58.24	262.48	49.57	39.84	587.69	
BGS	9.48	2.39	71.86	243.51	56.26	46.83	681.72	
Average	10.31	2.57	65.64	240.33	52.42	43.71	638.99	
National emission standard	75	20	1000	1500	200	1000	3000	GB 18918-2002
Agricultural standard limit of sludge (class A)	30	3	500	500	100	300	1500	GJ/T 309-2018
Landscaping limit (acid soil)	75	5	600	800	100	300	2000	GB/T 23486-2009
Mean in China		7.18	222	533	79.1	115	1270	[36]
Beijing	11.5	2.1	97.5	182.5	44.9	65.3	729.6	[38]
Guang Zhou		1.4	129.1	164.2	49.4	55.9	340.4	[14]
Hang Zhou	14.7	1.4	46.6	89.1	91.9	48	380.3	[39]
